# Acute Kidney Injury in HIV Infection

**DOI:** 10.4172/2329-891X.1000101

**Published:** 2013-02-25

**Authors:** Xuezhu Li, Shougang Zhuang

**Affiliations:** 1 Department of Nephrology, Shanghai East Hospital, Tongji University School of Medicine, Shanghai, China; 2 Department of Medicine, Rhode Island Hospital and Alpert Medical School of Brown University, USA

**Keywords:** Acute kidney injury, Human immunodeficiency virus, Nephropathy, Glomerulonephritis, HAART

## Abstract

Acute kidney injury (AKI) is increasingly recognized in clinical practice, and common in HIV-infection patients, affecting 18% of hospitalized patients. Preexisting hypertension, advanced HIV-infection, tenofovir toxicity, HCV co-infection, sepsis are risk factors of AKI. AKI can lead to prolonged hospitalization and is associated with increased mortality in HIV-infected patients. This review provides the most recent updates in the definition, diagnosis, pathophysiology, risk factors and treatment options for patients with HIV-associated AKI.

## Introduction

Renal disease is becoming an increasingly complication in populations with human immunodeficiency virus (HIV) infection [1]. HIV infection cannot only be turned into a chronic disease, named by HIV-associated nephropathy (HIVAN), but also increases the risk of acute kidney injury (AKI). AKI in HIV infected patients appears to be a risk factor for poor clinical outcomes, and associated with lengthened time of hospitalization and a high rate of mortality. Although HIVAN has been heavily studied in epidemiology, pathology and treatment, it is poorly addressed about HIV-associated AKI in these clinical aspects.

The Acute Kidney Injury Network (AKIN) defined AKI as the abrupt (≤ 48 hours) reduction of kidney function: increased serum creatinine levels (absolute, ≥ 0.3mg/dl; percentage, ≥ 50%; or 1.5 fold from baseline), or oliguria (< 0.5ml/kg/h for more than 6 hours) [2]. The occurrence of AKI increased length of hospital stay and excess health care costs [3]. To understand the importance of AKI in HIV-infected persons, this article will review recent developments in epidemiology, diagnosis, treatment and outcome of AKI in HIV-infected patients.

## Epidemiology of HIV Infection-Associated AKI

AKI is more common in HIV-infected individuals than their noninfected counterparts. Wyatt et al. [4], compared the incidence of AKI in HIV-infected patients in 1995 (pre- HAART (highly active antiretroviral therapy) era) and 2003 (post-HAART era) with the incidence of AKI being 2.9% and 6%, respectively. However, in a recent cohort study including 489 hospitalized HIV-infected patients, the incidence of AKI was 18% [5]. In another cohort of approximately 750 patients followed prospectively, more than 10% of patients developed at least 1 episode of AKI [6]. Recently, a retrospective study of all HIV positive children in Nigeria showed that ten children with kidney diseases, four were diagnosed as AKI and all of them died [7]. Thus, it seems that there is a huge variation of incidence of AKI between different studies due to the lack of widely accepted definition and of criteria AKI.

## Pathological Characteristics of HIV infection-associated AKI

**Figure 1:**
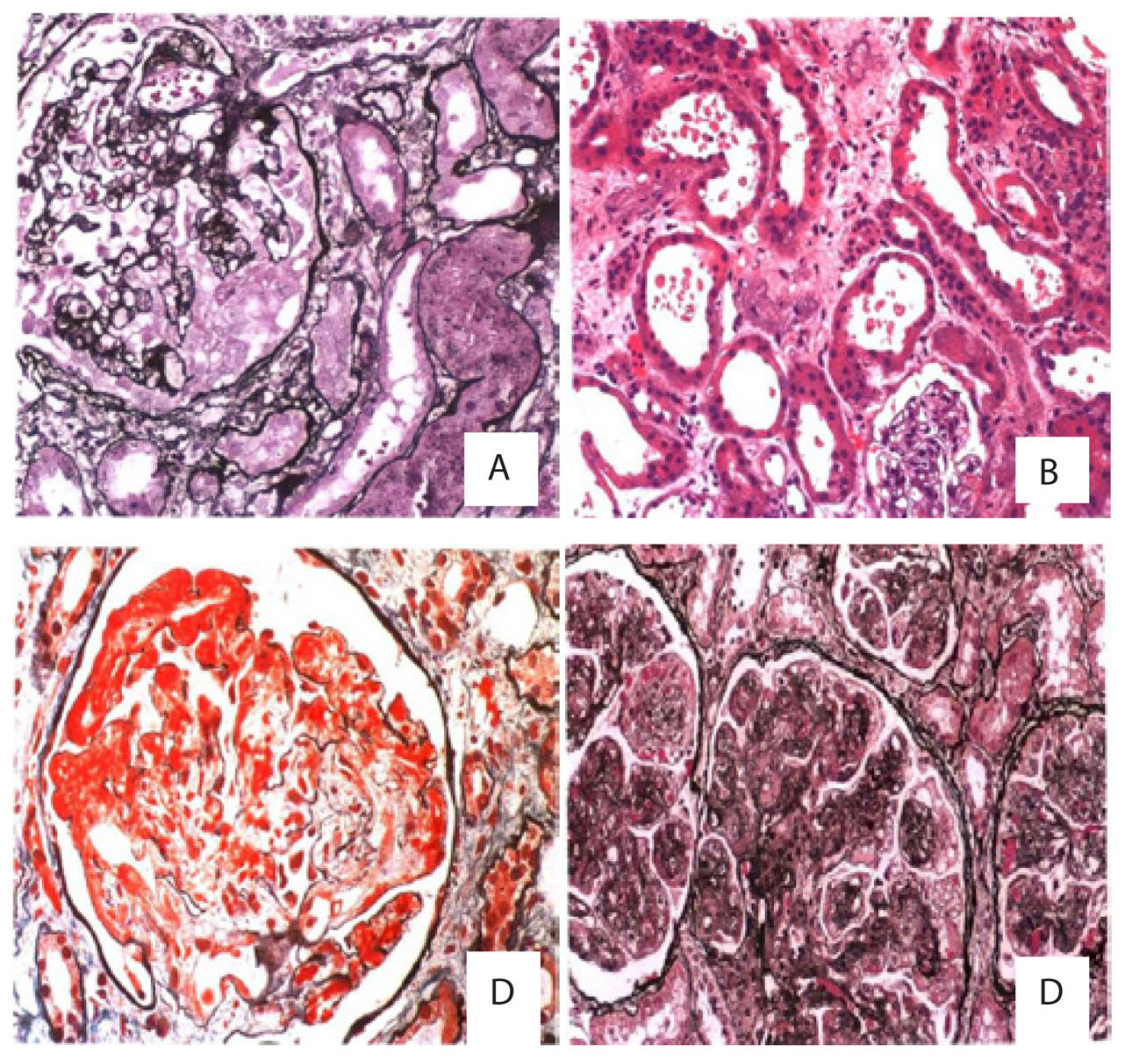
Pathological Characteristics of HIV infection-associated AKI A) HIV-associated nephropathy (HIVAN): lobal glomerular collapse with overlying epithelial cell crowding and hypertrophy; B) Acute tubular necrosis: Focal tubular necrosis at multiple points along the nephron and rupture of the basement membrane; C) Thrombotic microangiopathies: mesangiolysis, double contour basement membrane; D) membranoproliferative glomerulonephritis (MPGN): intense glomerular hypercellularity, diffuse thickening of the glomerular basement membrane with the appearance of “double contours”[14].

Wyatt et al. [4] reported that 84% of renal autopsy specimens from patients who died with HIV had evidence of histological renal disease, with the most frequently HIV-associated histopathologic manifestation: the collapsing variant of focal segmental glomerulosclerosis of HIV-associated nephropathy (HIVAN) which can manifest itself acutely or chronically. Acute tubular necrosis (ATN) and thrombotic microangiopathies (TMA) are also common pathological changes associated with AKI in HIV-infected patients [8] (Figure 1). ATN can be caused by ischemia and nephrotoxins, sepsis, congestive heart failure, or cirrhosis. Use of nephrotoxic medications increased the risk of ATN. These drugs include antibiotics, nonsteroidal anti-inflammatory drugs (NSAIDs), several antiretroviral medications (tenofovir and indinavir), or contrast media.

The incidence of TMA in HIV-infected patients has ranged from 0–7% in prospective and retrospective analyses [8,9], probably due to direct cytotoxic effects of HIV virus or opportunistic infection [10]. Peraldi et al., [11] found that thrombotic thrombocytopenic purpura (TTP) was the cause of approximately 50% of their biopsy cases of AKI in HIV-infected patients. Co-infection with HCV has been linked to several types of glomerular lesions including membranoproliferative glomerulonephritis (MPGN) and membranous glomerulopathy. A kidney biopsy should be considered in HIV-infected patients, although there is a longstanding perception that coagulopathies, thrombocytopenia and endothelial dysfunction in HIV-infected patients may increase the risk for bleeding, the most serious complications of biopsy [12]. However, a large retrospective study from The Johns Hopkins University School of Medicine did not confirm any increased risk of complications in HIV-infected individuals (8.6% in HIV-infected patients *versus* 7.2% in noninfected individuals) [13].

## Clinical characteristics of HIV infection-associated AKI

The Acute Kidney Injury Network defined AKI as the abrupt (≤ 48 hours) reduction of kidney function: increased serum creatinine levels (absolute, ≥ 0.3mg/dl; percentage, ≥ 50%; or 1.5 fold from baseline), or oliguria (<0.5ml/kg/h for more than 6 hours) [2]. The RIFLE (Risk, Injury, Failure, Loss, End-stage) criteria define AKI with three grades of increasing severity (Risk, Injury, Failure) and outlines two outcome variables – Loss and End-stage (Table 1). The RIFLE criteria have been validated in clinical settings for predicting patient outcomes [15]. A prospective cohort study of 56,823 HIV-infected patients in Veterans Affairs Medical Center showed that 82% of the patients who developed AKI were stage risk, 4.3% were stage injury, 6.8% were stage failure and 6.9% were AKI requiring dialysis [16]. Another retrospective study of 489 HIV-infected patients [5] showed that 36.4% of AKI were categorized as Risk, 34.1% as Injury and 29.5% as Failure. And the baseline serum creatinine was no difference according to RIFLE criteria. In the same study, the most common causes of AKI were sepsis (59%), nephrotoxic drug administration (37.5%), volume depletion (21.6%) and radiocontrast use (20.5%). AKI caused by infections usually presented as a prerenal disorder or ATN, while clinical presentation of AKI due to drug treatment was variable, including ATN, interstitial nephritis, crystalluria/obstruction, or prerenal disorder [6].

## Risk factors of HIV infection-associated AKI

### Chronic kidney disease (CKD)

The risk for AKI is significantly higher in HIV-infected patients with preexisting CKD than those who without CKD. In a prospective study, Li et al. [16] found that the risk for AKI was more than 5-fold greater for HIV patients with CKD (eGFR<60 ml/min/1.73m^2^) than for patients without CKD, while proteinuria (urine dipstick measurements greater than 30 mg/dl) conferred a 2-fold increase of risk for AKI. Ibrahim et al. [17] identified a 27-fold relative risk for AKI in patients with CKD compared with those with eGFR>90 ml/min/1.73m^2^. CKD is increasingly prevalent in HIV-infected patients. It has been shown that nearly half of CKD in HIV-infected patients was caused by HIVAN; other causes include immune complex disease, membranous/ membranoproliferative glomerulonephritis associated with viral hepatitis, diabetes and hypertension [18]. However, recent studies suggest that the spectrum of CKD in HIV-infected patients is changing with less HIVAN and more comorbid kidney disease, such as diabetes and hypertension [19]. And diabetic nephropathy and hypertensive nephrosclerosis are believed to be the leading causes of CKD in HIV-infected individuals [20]. As such, CKD screening is recommended for all the patients at the time of HIV diagnosis.

### Advanced HIV-infection

Several studies showed that advanced HIV-infection (measured by low CD4+ cell count and HIV viral load) was an independent risk factor for AKI during the highly active antiretroviral therapy (HAART) era and the pre-HAART era [6]. Roe et al. [21] found that AKI was associated with low CD4 count (<100 cells/mm^3^), Ibrahim et al. [17] found that lower levels of CD4 (<200 cells/mm^3^) were strongly associated with higher AKI risk. These data showed that immunodeficiency is a potent risk factor for AKI.

### Antiretroviral drugs toxicity

Tenofovir, a nucleotide reverse transcriptase inhibitor, is one of the most commonly used medications in HAART therapy. However, it has been reported to be associated with 0.5–5% risk of AKI in HIV positive patients. Approximately 2% of patients taking tenofovir develop a proximal tubulopathy, which can cause Fanconi’s syndrome before leading to ATN. One meta-analysis demonstrated that HIV seropositive patients receiving tenofovir had a mean difference of 3.9 ml/min in eGFR, compared with those who did not receive this drug [22]. A report from the EuroSIDA cohort in 2010 indicated that cumulative exposure (upto more than 3 years) to tenofovir, indinavir, and ritonavir-boosted lopinavir was associated with a small but statistically significant increase (*P*<0.0001) in risk of decline of renal function (eGFR<60mL/min) [23]. A recent cohort study indicated that Tenofovir-induced AKI developed in 25 of 512 patients (4.88%). The average time for developing AKI was 6 months. On stopping tenofovir, 15 patients had complete recovery of renal function, 5 had partial recovery while 5 patients died [24].

The mechanism of tenofovir renal toxicity is still unclear, but may be associated with single nucleotide polymorphisms in renal transporters, multidrug resistance-associated protein 2 (MRP2) [20]. And Tenofovir dose adjustment is recommended when estimated GFR <60ml/min/1.73m^2^ [25]. Thus, although tenofovir is an important drug for antiretroviral therapy, more attention is being paid to potential renal toxicity in clinical use. Early detection of proximal-tubular injury-glucosuria with normal serum glucose, hypophosphatemia or hypokalemia with increased urinary excretion–is recommended to prevent nephrotoxicity by adjusting tenofovir dose or switching to other antiretrovirals. Indinavir may promote crystalluria, obstructive uropathy or crystal nephropathy. Microscopy of the urine sediment reveals crystals of varying shapes, including plate-like rectangles, fanshaped crystals, and star bust forms [26], which can be distinguished from tubular toxicity damage. Other antiretroviral drugs may also cause kidney injury, (Table 2).

Several studies [1,4,11,16–17,19–20] have also analyzed the other risk factors for AKI in HIV-infected patients, including HCV coinfection, low serum albumin (<3.7 mg/dl), low body mass index (<18.5 kg/m^2^), black race, hypertension, diabetes, cardiovascular disease, hypomagnesemia, male gender and older age [27]. The risk factors are clinical predictors for AKI and outcomes.

## Treatment of HIV-associated AKI

Treatment of patients with HIV-associated AKI is varied and depends on etiologic factors. Prerenal azotemia caused by volume depletion is usually responsive to administration of fluids and electrolytes, maintenance of hemodynamics, adjustment for acidosis and electrolyte abnormalities. Drug-induced AKI requires stopping of nephrotoxic agents. Because the absence of effective pharmacotherapeutic options for AKI, renal replacement therapy (RRT) is as the predominant component of care in patients with severe AKI.

Hemodialysis (HD) was the most frequently used type of dialysis than peritoneal dialysis (PD) in HIV-infected patients [28]. In the United States, the number of HIV-infected patients receiving dialysis in dialysis units keeps increasing from 1985 (0.3%) to 1992 (1.5%) [29]. In 2002, 1.5% of patients receiving dialysis had HIV infection and 0.4% had AIDS, respectively [30]. Complications in HIV-infected patients receiving HD were frequent: one-third of the patients had cardiovascular disease and one-fourth had malignancies [31]. Some researchers have proposed HD, by causing leukocyte activation and cytokine release, could actually accelerate HIV replication [32]. Standard infection control procedures should be followed to prevent the spread of HIV within a hemodialysis center. The infection of health care workers with HIV by needle stick injury is much less than with hepatitis B and C [33]. Isolation of HIV-infected patients or use of separate HD machines is not required on the base of standard disinfection and sterilization procedures. Since HIV virus has not been isolated from HD ultrafiltrate, the CDC does not prohibit participation of HIV-infected patients in hemodialyzer reuse programs [34].

The incidence of *Staphylococcus aureus* peritonitis in HIV-infected patients receiving continuous ambulatory PD is dramatically higher than HIV-negative continuous ambulatory PD controls, and HIV-infected patients had a 50% increased risk of peritonitis and a 3-fold increased risk of death [35]. Replication-capable HIV has been found in PD tubing and effluent bags for up to 7 days, so PD fluid effluent should be treated for at least 30 minutes with bleach and fluid, tubing, and bags disposed of as biohazard waste [36]. HIV positive patients with renal complications should be referred to a nephrologist. Screening for AKI should be carried out, serum creatinine, phosphate, urine alpha-1-microglobuline and proteinuria should be monitored for HIV infected patients, in particular, when they are receiving antiretroviral drugs with nephrotoxicity.

## Outcomes

AKI is associated with poor health outcomes in HIV-infected patients. In a cohort study [4], in-hospital mortality of AKI in HIV-infected patients was 6-fold higher than seen in admissions of HIV-infected patients without AKI (27% *vs.*4.5%). Even an asymptomatic increase in serum creatinine can lead to an increased risk of heart failure, cardiovascular events, end stage renal disease (ESRD) and death [37]. Hospitalizations of HIV-infected patients that were complicated by AKI were also complicated by much higher in-hospital mortality (27%) than seen in admissions of HIV-infected patients without AKI (4.5%) [5].

In a cohort study, the cumulative probability of death of patients with AKI at 6 months, 1 and 2 years of follow-up was 8.3, 16.9 and 34.2%, respectively [38]. Tourret et al. [39] enrolled 164 HIV positive hemodialysis patients in a prospective cohort, showed that the 1 year survival rate was 93.8 ± 1.9%, and the 2 year survival rate was 89.4 ± 2.4% [39]. A multicenter retrospective cohort study showed that survival rates at 1, 3, and 5 years for HIV infected hemodialysis patients were 95.2%, 71.7%, and 62.7%. Medium-term survival of HIV-infected patients on dialysis was lower than that of matched HIV-negative patients. Five-year survival for HIV-negative patients was 94.4% [40]. Dialysis modality was not a factor in survival of patients with HIVAN. Ahuja et al. [41] compared survival between 5,299 patients who received hemodialysis and 716 patients who received peritoneal dialysis in the USA, found that there was no difference in survival between the different modalities (hazard ratio: peritoneal dialysis vs. hemodialysis, 1.04, 95% CI, 0.96–1.13).

## Conclusion and Perspective

AKI is a common complication in HIV-infected patients and has been associated with prior renal impairment. HIV-infected patients are also at increased risk for AKI during hospitalization, due to volume depletion, sepsis and the acute administration of nephrotoxic medications or radiocontrast. Although HAART has improved long-term outcomes of HIV-infected patients, mortality of AKI in HIV-infected patients is still high. Currently, there is no specific treatment for HIV-associated AKI, prophylaxis should be considered for any patients with classic risk factors for AKI. Future research should focus on elucidation of pathophysiology of HIV-associated AKI, and development of new therapeutic strategies for preventing AKI in HIV-infected patients.

## Figures and Tables

**Table 1:** The RIFLE criteria for acute kidney injury.

Class	Increase in Glomerular Filtration Rate	Reduced Urine Output by Symptom Duration

**Severity**		
Risk	1.5-fold	<0.5 ml/kg/h for 6 h
Injury	Two fold	<0.5 ml/kg/h for 12 h
Failure	Three fold*	<0.3 ml/kg/h for 24 h†

**Outcome** Loss –Persistent acute kidney injury with complete loss of function for more than 4 wk End-stage kidney disease for more than 3 month		

* A threefold increase in serum creatinine or a serum creatinine level ≥ 4.0mg/ dL with an acute rise ≥ 0.5 mg/dL indicates renal failure.

† Likewise, anuria for 12 hours indicates renal failure.

**Table 2:** Nephrotoxicity of antiretroviral drugs.

Drugs	kidney injury
Tenofovir	Proximal tubulopathy with Fanconi syndrome
	ATN
Indinavir	crystal associated nephropathies
Atazanavir	AIN
	Nephrolithiasis
Ritonavir	AKI
Efavirenz	Nephrolithiasis
T20	glomerulopathy
Didanosine (ddI)	accelerated mitochondrial toxicity
Stavudine (d4T)	ATN
Lamivudine (3TC)	Fanconi syndrome, nephrogenic diabetes insipidus
Ganciclovir	Crystal nephropathy
Adefovir	ATN, mitochondria injury
Pentamidin	acute tubular necrosis
NSAID	Proteinuria, secondary minimal change disease, papillary necrosis
